# Using local scale exponent to characterize heart rate variability in response to postural changes in people with spinal cord injury

**DOI:** 10.3389/fphys.2015.00142

**Published:** 2015-05-12

**Authors:** Fuyuan Liao, Ben-Yi Liau, Ian M. Rice, Jeannette Elliott, Ian Brooks, Yih-Kuen Jan

**Affiliations:** ^1^Rehabilitation Engineering Laboratory, Department of Kinesiology and Community Health, University of Illinois at Urbana-ChampaignChampaign, IL, USA; ^2^Department of Biomedical Engineering, Xi'an Technological UniversityXi'an, China; ^3^Department of Biomedical Engineering, Hungkuang UniversityTaichung, Taiwan; ^4^Division of Disability Resources and Educational Services, University of Illinois at Urbana-ChampaignChampaign, IL, USA; ^5^National Center for Supercomputing Applications, University of Illinois at Urbana-ChampaignUrbana, IL, USA

**Keywords:** complexity, heart rate variability, local scale exponent, spinal cord injury

## Abstract

Heart rate variability (HRV) is a promising marker for evaluating the remaining autonomic function in people with spinal cord injury (SCI). HRV is commonly assessed by spectral analysis and detrended fluctuation analysis (DFA). This study aimed to investigate whether local scale exponent α(t) can reveal new features of HRV that cannot be reflected by spectral measures and DFA coefficients. We studied 12 participants with SCI and 15 healthy able-bodied controls. ECG signals were continually recorded during 10 min sitting and 10 min prone postures. α(t) was calculated for scales between 4 and 60 s. Because α(t) could be overestimated at small scales, we developed an approach for correcting α(t) based on previous studies. The simulation results on simulated monofractal time series with α between 0.5 and 1.3 showed that the proposed method can yield improved estimation of α(t). We applied the proposed method to raw RR interval series. The results showed that α(t) in healthy controls monotonically decreased with scale at scales between 4 and 12 s (0.083–0.25 Hz) in both the sitting and prone postures, whereas in participants with SCI, α(t) slowly decreased at almost all scales. The sharp decreasing trend in α(t) in controls suggests a more complex dynamics of HRV in controls. α(t) at scales between 4 (0.25 Hz) and around 7 s (0.143 Hz) was lower in subjects with SCI than in controls in the sitting posture; α(t) at a narrow range of scales around 12 s (0.083 Hz) was higher in participants with SCI than in controls in the prone posture. However, none of normalized low frequency (0.04–0.15 Hz) power, the ratio of low frequency power to high frequency (0.15–0.4 Hz) power and long-term (>11 beats) DFA coefficient showed significant difference between healthy controls and subjects with SCI in the prone posture. Our results suggest that α(t) can reveal more detailed information in comparison to spectral measures and the standard DFA parameters.

## Introduction

Spinal cord injury (SCI) disrupts the descending autonomic pathways, resulting in a variety of dysfunction of the cardiovascular system related to the level and severity of injury to these pathways (Alexander et al., 2009). This commonly manifests as neurogenic shock, orthostatic hypotension, autonomic dysreflexia, and cardiac rhythm disturbances (Claydon and Krassioukov, 2006; Krassioukov and Claydon, 2006). To date, the assessment to quantify severity of injury to autonomic pathways has not been well developed, and there is still no consensus on this issue (West et al., 2013). The clinical practice focuses on the assessment of motor and sensory impairments following SCI according to the International Standards for the Neurological Classification of SCI, rather than assessment of severity of injury to autonomic pathways (Claydon and Krassioukov, 2008; Jan et al., 2013).

Heart rate variability (HRV) represents one of the most promising markers of autonomic activities (Task Force of the European Society of Cardiology and the North American Society of Pacing and Electrophysiology, 1996). It has been widely accepted that fluctuations in heart rate (HR) occurring at different frequencies reflect the activities of autonomic neural outflows (West et al., 2013). Previous research has shown great potential of HRV for evaluating the remaining autonomic functions of the cardiovascular system in patients with SCI (Merati et al., 2006; Claydon and Krassioukov, 2008; Jan et al., 2013). Various methods have been developed to quantify HRV, including time domain, frequency domain, and nonlinear methods (Task Force of the European Society of Cardiology and the North American Society of Pacing and Electrophysiology, 1996). The power spectra density (PSD) of HRV reveals two characteristic frequencies: one is defined as the low frequency (LF, 0.04–0.15 Hz) and the other is defined as the high frequency (HF, 0.15–0.4 Hz). HF of HRV reflects vagal modulation, and LF is considered to be jointly mediated by sympathetic and vagal nerves (Task Force of the European Society of Cardiology and the North American Society of Pacing and Electrophysiology, 1996). The ratio of LF to HF power is widely used as an index of sympathovagal balance for assessing cardiovascular regulation. Indeed, time and frequency domain indexes of HRV have provided prognostic information in many pathological conditions. However, in addition to autonomic modulations, HRV can also be influenced by other mechanisms, e.g., intrinsic variations of pacemaker rate (Ponard et al., 2007) or fluctuations in circulating neurohumoral factors (Galetta et al., 2008). These mechanisms may operate at time scales that time and frequency domain methods cannot capture adequately (Tan et al., 2009). Hence, scale independent measures have been introduced to study the integrated control of HR. The use of such measure is based on the observation that HR fluctuations exhibit scale-invariant patterns over a wide range of time scales that breakdown under pathological conditions (Peng et al., 1995; Goldberger et al., 2002).

Detrended fluctuation analysis (DFA) (Peng et al., 1995) is one of the most commonly used scale independent method. DFA provides a quantitative parameter, the scaling exponent α, to represent the correlation properties of the data (Peng et al., 1995). Usually, the properties of HRV data (RR interval series) are described by two scaling exponents: a short-term (4–11 beats) exponent α_1_ and a long-term (>11 beats) exponent α_2_ (Beckers et al., 2006). It was demonstrated that α_1_ of HRV can reflect changes in autonomic tone induced by exercise, maneuvers such as passive head-up tilt, cold hand immersion, cold face immersion (Tulppo et al., 2001, 2005), and aging or cardiac pathologies (Beckers et al., 2006). A growing number of studies have suggested that α_1_ can yield prognostic information that cannot be provided by conventional measures (Huikuri et al., 2009). However, the use of a single scaling exponent for characterizing the HR dynamics has been questioned (Ivanov et al., 1999; Echeverria et al., 2003; Castiglioni et al., 2009, 2011). It has been recognized that HRV does not always present a uniform power-law behavior, especially in abnormal physiological conditions (Echeverria et al., 2003). Ivanov et al. (1999) suggested that human heartbeat dynamics requires a large number of exponents to characterize its properties. A recent study even suggested that the physiological effects of autonomic outflow may mask intrinsic fractal behavior of the sinoatrial node (Tan et al., 2009). Some authors therefore proposed to use a whole spectrum of local scale exponents α(*t*) to describe HR dynamics (Bojorges-Valdez et al., 2007; Castiglioni et al., 2009, 2011), where *t* is the time scale. Castiglioni et al. (2011) suggested that α(*t*) describes the local correlation properties of data. By using autonomic blocking agents, they showed that both cardiac sympathetic and vagal outflows affect the spectrum of α(*t*) but with opposite effects. However, it is still unclear whether the spectrum of α(*t*) reveals new features that are not characterized by the PSD and DFA coefficients. In addition, α(*t*) at small scales can be overestimated (Viswanathan et al., 1997; Kantelhardt et al., 2001). This problem was not well addressed in the previous studies (Castiglioni et al., 2009, 2011). This is an important issue because α(*t*) at small scales and their mean value (i.e., α_1_), have been used to assess HR dynamics due to their sensitivity to changes in cardiac autonomic tone (Tulppo et al., 2001, 2005).

The purpose of this study was to investigate whether α(*t*) reveals new features of HRV in people with SCI that cannot be reflected by spectral measure and DFA coefficients. This was done by changing the posture from the sitting to prone posture in people with SCI, which is known to induce autonomic modulated vasodilatory responses. To accurately estimate α(*t*), we developed an approach for correcting α(*t*) at small scales based on a previous study by Kantelhardt et al. (2001). Our method may be used to evaluate the local properties of HRV and may enhance the current understanding of residual autonomic function in people with SCI.

## Methods

### Subjects

Twenty seven participants were recruited in this study, including 12 people with SCI and 15 healthy able-bodied controls. Their demographic data are shown in Table 1. The SCI group included five participants with a spinal injury level at C4–T5 [five incomplete, American Spinal Injury Association Impairment Scale (AIS) B, C, or D] and seven subjects with a spinal injury level at T6–T12 [four complete (AIS A) and three incomplete (AIS B, C, or D)]. All participants with SCI were in a stable clinical condition (the injury event occurred more than 6 months before the time of the study). None of the subjects had any diagnosed cardiovascular or neurological diseases that might affect autonomic cardiovascular control. All participants gave informed consent to participate in this study, which was approved by the Institutional Review Board of the University of Oklahoma Health Sciences Center.

**Table 1:** **Demographic data of the enrolled subjects**.

	**SCI**	**Controls**
Number of subjects	12	15
Gender, M/F	9/3	11/4
Age, yr	35.1 ± 11.9	29.4 ± 6.2
Body mass index, kg/m^2^	25.8 ± 4.9	24.3 ± 2.9
Duration of injury, yr	7 (4, 12)	/
Number of C4–T5/T6–T12	5/7	

*Data are expressed as mean ± standard deviations or median (25th, 75th percentiles)*.

### Data collection

All experiments were performed in a university laboratory. Room temperature was maintained at about 23°C. Each subject was asked to stay in the laboratory for at least 30 min to acclimate to the room temperature. When a subject sat in the wheelchair, the electrocardiography (ECG) electrodes of a three-lead Biopac ECG monitor (ECG100C, Biopac Systems; Goleta, California) were placed on the right ventral wrist, right medial ankle, and left medial ankle. The ECG signals were recorded for 10 min with a sampling rate of 1000 Hz. Then the subject was transferred to a mat table in a prone position for 10 min recording. Able-bodied controls followed the same procedures but changed from the sitting posture to the prone posture without assistance. By using the AcqKnowledge software (Biopac Systems), the ECG signal was edited to remove artifacts and then filtered with a bandpass filter of 0.5–32 Hz. After RR peak detection and visual inspection by the operator, the consecutive RR intervals (RRIs) were exported for later processing.

### Linear analysis

Mean and standard deviation (SD) of RRI series were calculated in the time domain. Then the RRI series was resampled at 2 Hz using a cubic spline approximation (Aubert et al., 1999). The PSD of HRV was calculated using an autoregressive model with afixed order of 16 (Boardman et al., 2002). Three parameters of PSD were calculated according to the published guideline (Task Force of the European Society of Cardiology and the North American Society of Pacing and Electrophysiology, 1996): normalized low frequency power [% LF = 100×LF power/(total power-VLF power)], high frequency power, and LF to HF ratio (LF power/HF power), where total power was defined as the variance of the data series and VLF was the very low frequency range (<0.04 Hz).

### Detrended fluctuation analysis (DFA)

The DFA method applied to RRI series has been described elsewhere (Peng et al., 1995). We have briefly described the methods used in this study here. Given a series of *N* RRIs, {*x*(*i*), *i* = 1,…,*N*}, it was first integrated after mean subtraction

(1) $$y\left(k\right)=\sum_{i=1}^{k}\left(x\left(i\right)-\langle RR\rangle \right),$$

where 〈*RR*〉 was the mean of the series. Next, the integrated series *y*(*k*) was divided into boxes of *n* RRIs. In each box, the local trend, *y*_*n*_(*k*), was estimated by a least-square fit of *y*(*k*) using a polynomial function (a linear function was used throughout this work). The root-mean square fluctuation

(2) $$F\left(n\right)=\sqrt{\frac{1}{N}\sum_{k=1}^{N}\left({\left(y(k)-y_{n}\left(k\right)\right)}^{2}\right)}$$

was calculated for box sizes *n*≥ 4. A power-law between *F*(*n*) and *n* indicated the presence of scaling: *F*(*n*)~ *n*^α^. The parameter α, called scaling exponent, was estimated by the slope of the *log*[*F*(*n*)]–*log*(*n*) plot. α represents the correlation properties of the signal. For examples, α = 0.5 indicates white noise, α = 1 represents the behavior of a 1/*f* process having persistent long range correlations, and α = 1.5 indicates Brownian noise. For HRV data, standard DFA typically includes a short-term (4–11 beats) scaling exponent α_1_ and a long-term (>11 beats) scaling exponent α_2_ (Beckers et al., 2006).

### Local scale exponent

A local scale exponent was defined as the derivative of log[*F*(*n*)] with respect to log(*n*)

(3) $$\alpha (n_{k})=\frac{log\left[F(n_{k+1})\right]-log[F(n_{k-1})]}{log(n_{k+1})-log(n_{k-1})},$$

where {*n_k_*} was a set of box sizes spaced evenly on a log scale. An intrinsic problem was that the *log*[*F*(*n*)] − *log*(*n*) plot deviates from scaling at small scales, resulting in over-estimations of scale exponents at small scales (Viswanathan et al., 1997; Kantelhardt et al., 2001; Echeverria et al., 2003). To address this problem, Kantelhardt et al. (2001) introduced a correction function

(4) $$K^{\alpha }(n)=\frac{{\langle {\left[F(n)\right]}^{2}\rangle }^{1/2}/n^{\alpha }}{{\langle {\left[F(n\prime )\right]}^{2}\rangle }^{1/2}/n\prime^{\alpha }},$$

where 〈·〉 denotes the average over different configurations, and *n*′ is a specific box size. Because *K*^α^(*n*) depends only weakly on α, *K*^α^(*n*) for uncorrelated data, i.e., *K*^1/2^(*n*), can be used in all cases. *K*^1/2^(*n*) can be obtained by analyzing the corresponding shuffled data, where all long-range correlations are destroyed by shuffling. Kantelhardt et al. (2001) suggested that *n*′ has to be large (*n*′ > 50) but must remain significantly smaller than the record length *N* and they suggested *n*′ ≈ *N*/20 to be a reasonable number. The modified fluctuation function is given by Kantelhardt et al. (2001)

(5) $$F_{mod}\left(n\right)=F(n)/K^{1/2}(n).$$

**Figure 1:**
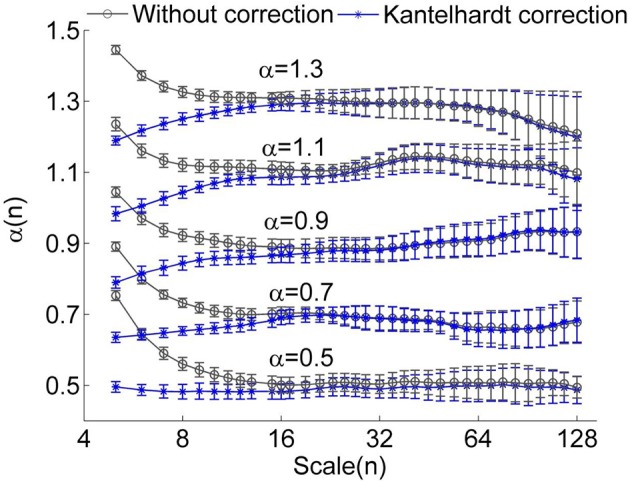
**Local scale exponents α(*n*) for monofractal time series with α between 0.5 and 1.3**. The monofractal time series were generated by inverse Fourier transform of the power spectrum *S*(*f*) ∝ *f*^−β^ with β = 2α −1. When calculating α(*n*), linear trends were removed. The results were obtained by averaging over 50 series having a length of 750 data points. The circles represent the values without correction and the stars represent the values corrected using the method proposed by Kantelhardt et al. (2001). The error bars represent the standard deviations.

**Figure 2:**
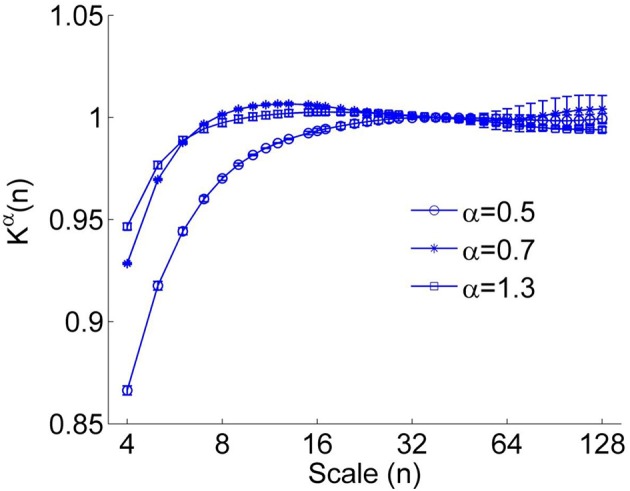
**The correction function *K*^α^(*n*) for α = 0.5, 0.7, and 1.3. A larger value of α leads to more prominent deviation between *K*^α^(*n*) (Equation 4) and *K*^1/2^(*n*)**. The error bars represent the standard deviations.

However, we found that for short time series, this method could result in underestimated α(*n_k_*) at small scales even for using a linear function *y_(k_*) when calculating the fluctuation function *F*(*n*) (Figure 1). Furthermore, it seems that a larger value of α leads to more prominent bias. The reason is that a larger value of α leads to more prominent differences between *K*^α^(*n*) (Equation 4) and *K*^1/2^(*n*) at small scales (see Figure 2). Thus, the underestimation bias in α(*n*) can be corrected as

(6) $$\alpha \left(n\right)=\alpha_{K}(n)\cdot K^{\alpha }(n)/K^{\frac{1}{2}}(n),$$

where α*_K_(n)* denotes local scale exponents obtained by using the method by Kantelhardt et al.

**Figure 3:**
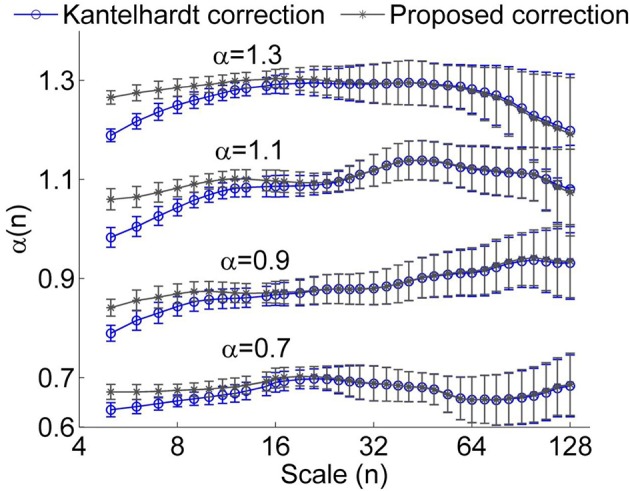
**Local scale exponent α(*n*) for monofractal time series obtained by using the method proposed by Kantelhardt et al. (Kantelhardt correction) and by the proposed method**. In these calculations, linear trends were removed. The results were obtained by averaging over 50 series having a length of 750 data points. The error bars represent the standard deviations.

To verify whether the proposed method improves the estimation of α(*n*), we considered simulated monofractal time series with α between 0.5 and 1.3, which were generated by inverse Fourier transform of the power spectrum *S*(*f*) ∝ *f*^−β^ with β = 2α −1. In Figure 3, the values of α(*n*) obtained by using Equation (6) are compared to those obtained by using Equation (5). Obviously, the suggested method substantially improves estimations of α(*n*) at small scales. Since for RRI series, the mean value of α(*n*) at *n* < 11 beats, i.e., the short-term DFA coefficient α_1_, is generally larger than 1 (Beckers et al., 2006), we argue that the proposed method can yield improved results.

**Figure 4:**
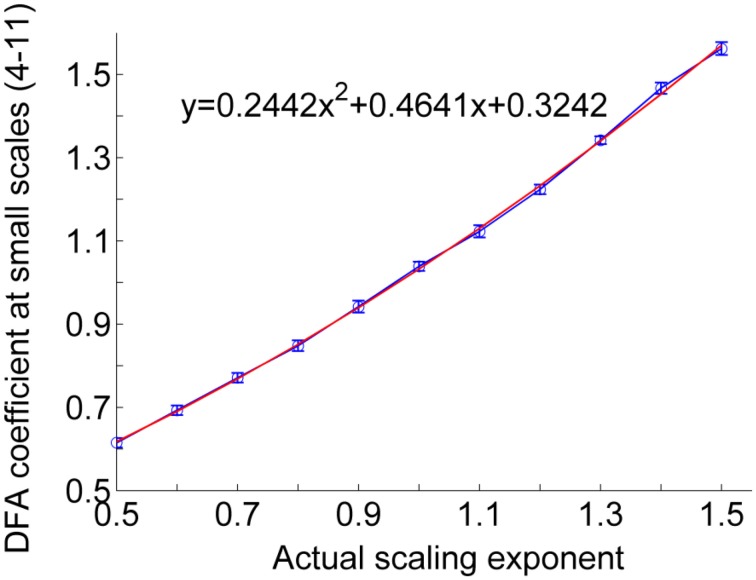
**Relationship between actual scaling exponent α and DFA coefficient at scales between 4 and 11**. The circles represent the mean values of DFA coefficients of 50 simulated monofractal signals with a length of 750 points. The lengths of the error bars represent the standard deviations. The fitting curve (red line) was computed in a least-squares sense.

For a RR interval series, the procedure of the proposed correction method is as follows. (1) Calculation of the short-term DFA coefficient α1 of the RR interval series. In general, the scales for calculating α_1_ range between four beats and a specific scale around 11 beats. (2) Estimation of the actual scaling exponent α from α_1_. Figure 4 shows the relationship between the actual exponent α and α_1_ for monofractal time series with α between 0.5 and 1.5, where the monofractal time series of the same length of the RR interval series can be generated by inverse Fourier transform. According to this relationship, i.e., the fitting curve (in red color), it is easy to estimate α from α_1_. (3) Calculation of local scale exponents using the method proposed by Kantelhardt et al. (4) Calculation of local scale exponent using Equation (6).

### Application to HRV data

Because the time duration of a box size depends on the mean RRI, if two series have different values of mean RRI, the same box size actually represents different time scales. This is the case in many studies under different experimental conditions. In this study, mean RRI was generally shorter in the sitting posture compared to the prone posture, especially in able-bodied controls. In order to compare scale exponent spectrum of HRV between the sitting and prone postures and among different subjects, we converted the box sizes {*n_k_*} to time scales {*t_k_*} by Castiglioni et al. (2009)

(7) $$t_{k}=n_{k}\times \;\langle RR\rangle .$$

Then the spectrum α(*t_k_*) was linearly interpolated to obtain a new spectrum α(*t_i_*) with *t_i_* evenly distributed between 4 and 60 s on a log scale.

The rationales for choosing the time scale interval are as follows. In DFA of RRI series, the smallest box size is usually chosen as four beats (Beckers et al., 2006). For most of our data sets, mean RRI ranges between 0.6 and 1 s. Thus, the time scale corresponding to the smallest box size of four RRIs ranges between 2.4 and 4 s. We therefore chose 4 s as the smallest time scale. In the previous studies by Castiglioni et al. (2009, 2011), the smallest time scale was also chosen as 4 s. On the other hand, the largest time scale depends on the length of data series (10 min). Kantelhardt (2009) suggested that the largest scale can be *N*/4. Castiglioni et al. (2011) reported that α(*n*) can be estimated without substantial bias up to scales equal to *N*/7. In their study, the largest time scale for 15–20 min record was chosen as 100 s. However, our simulation results on monofractal time series indicated that, when scales exceed *N*/20, α(*n*) may become unstable and deviate from the expected values (Figures 1, 3). Thus, we speculate that for RRI series derived from 10 min ECG signals, the reliability of estimations of α(n) decreases when time scale exceeds 30 s. The time scale range 4–30 s corresponds to the frequency interval 0.033–0.25 Hz. It has been well known that in spectral analysis of HRV, the low frequency (LF) and high frequency (HF) bands are defined as 0.04–0.15 Hz and 0.15–0.4 Hz, respectively. According to the published guidelines (Task Force of the European Society of Cardiology and the North American Society of Pacing and Electrophysiology, 1996), for short-term recordings (~5 min), assessment of very low frequency, i.e., <0.04 Hz, should be avoided. In this study, we also presented α at scales between 30 and 60 s just for reference.

### Statistical analysis

The Wilcoxon signed-ranked test was used to examine the differences of linear measures of HRV, scaling exponents α_1_and α_2_, and local scaling exponent α(*t*) between sitting and prone postures (within-subjects test). The Wilcoxon rank-sum test was used to examine the differences in these measures between patients with SCI and able-bodied controls (between-subjects test).

## Results

### Linear parameters

**Figure 5:**
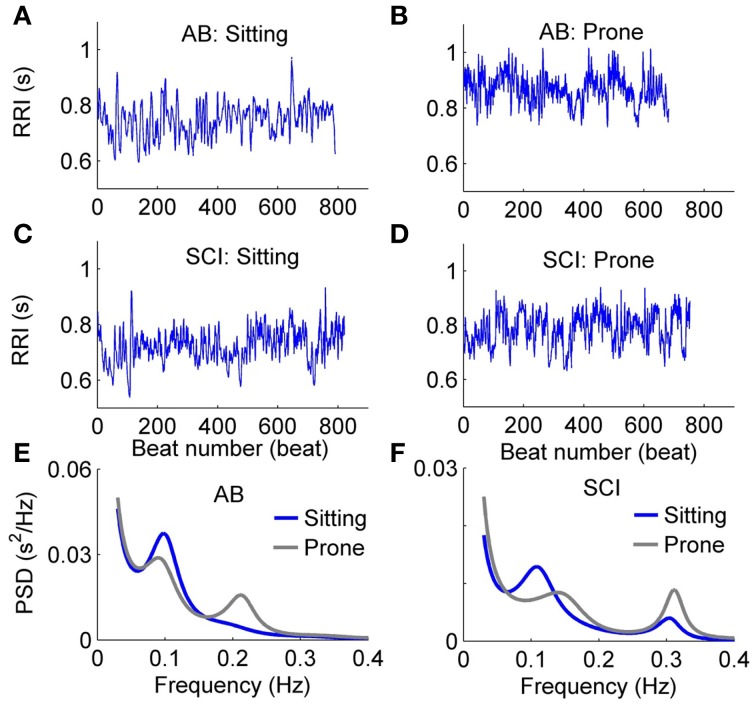
**Typical examples of changes in RRI and PSD of HRV in response to the postural change in an able-bodied control (A,B,E) and a patient with SCI (C,D,F)**.

Figure 5 shows typical examples of changes in RRI and PSD of HRV in response to the postural change in a healthy able-bodied control (Figures 5A,B,E) and a person with SCI (Figures 5C,D,F). In the control, RRI showed a distinctive increase after the change from the sitting to prone posture (Figures 5A,B). However, RRI in the person with SCI showed only a slight increase after the postural change (Figures 5C,B). The PSD of HRV in the control revealed a decrease in LF power and an increase in HF power (Figure 5E), whereas the PSD of HRV in the person with SCI showed only slight changes (Figure 5F).

Table 2 compares the linear parameters between healthy able-bodied controls and subjects with SCI. Mean RRI was lower in the sitting position in both groups. In controls, %LF was significantly higher, HF power was significantly lower, and thus LF/HF was significantly higher in the sitting posture, whereas in subjects with SCI, these parameters did not show significant change after the postural change. Compared to controls, %LF was significantly lower, HF power was significantly higher, and LF/HF was significantly lower in subjects with SCI in the sitting position. No significant difference was observed between two groups in the prone position.

**Table 2:** **Linear parameters of HRV in healthy able-bodied controls and subjects with SCI**.

**Position**	**Mean RRI (ms)**	**SD of RRI (ms)**	**% LF**	**HF power (ms^2^)**	**LF/HF**
**ABLE-BODIED CONTROLS**					
Sitting	799.7±39.3^*^	70.1±7.7	71.3±3.8^*^	638±200^*^	3.3±0.8^*^
Prone	916.2±46.0	83.6±13.9	42.0±4.9	1193±157	0.8±0.2
**SCI**					
Sitting	770.9±26.0^*^	62.7±19.5	53.5±5.2^+++^	359±109^+^	1.6±0.4^+++^
Prone	826.1±26.6	71.4±23.5	49.2±4.7	720±216	1.3±0.2

*Values are means ± SE*.

* p < 0.05 vs. prone;

+ p < 0.05 vs. healthy able-bodied controls;

+++ *p < 0.001 vs. healthy able-bodied controls*.

### DFA coefficients

**Figure 6:**
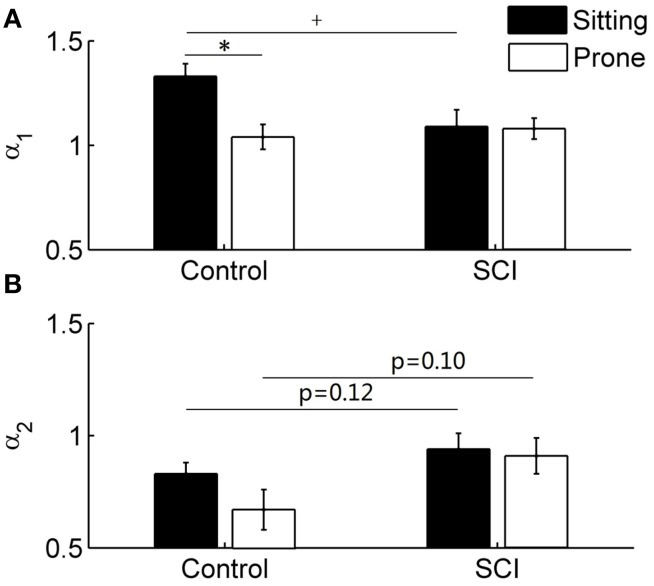
**DFA coefficients α_1_ (short-term) and α_2_ (long-term) in able-bodied controls and subjects with SCI**. Values are means ± SE. ^*^p < 0.05 for within-subjects test; ^+^*p* < 0.05 for between-subjects test. **(A)** α_1_ in control and SCI groups. **(B)** α_2_ in control and SCI groups.

The short-term scaling exponent α_1_was significantly higher in the sitting posture than in the prone posture in healthy able-bodied controls (*p* < 0.05) but not in subjects with SCI (Figure 6). In the sitting posture, α_1_ was significantly higher in controls than in subjects with SCI (Figure 6A). Unlike α_1_, the long-term scaling exponent α_2_ showed only slight changes after the postural change in either control or SCI group (Figure 6B) and no significant differences were observed between two groups (in the sitting posture, *p* = 0.12; in the prone posture, *p* = 0.10).

### Local scale exponent

**Figure 7:**
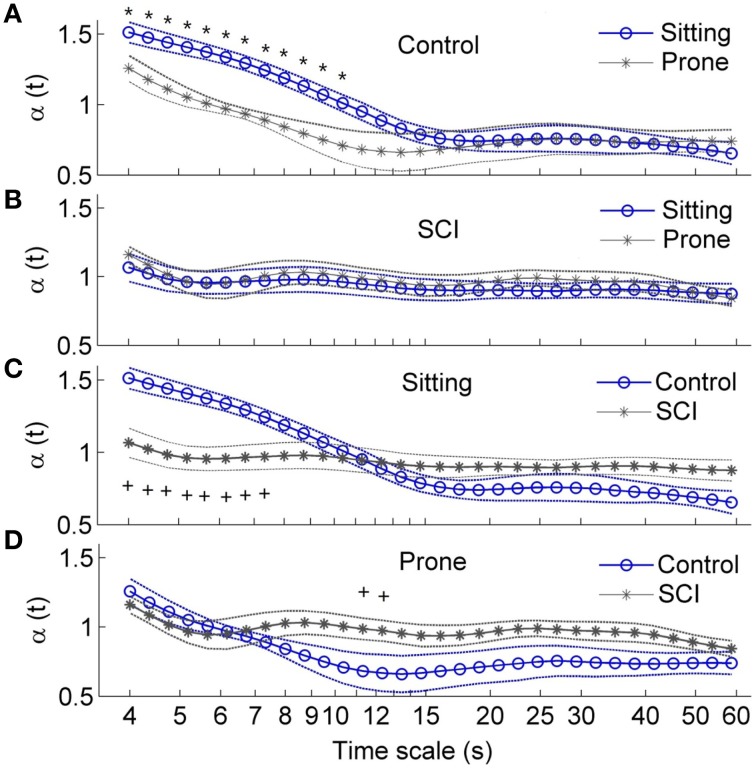
**Local scale exponents α*t* in healthy able-bodied controls and subjects with SCI**. Values are presented as means ± SE. ^*^p < 0.05 for within-subjects test; ^+^*p* < 0.05 for between-subjects test. **(A)** Comparisons of α(*t*) in control group between two postures. **(B)** Comparisons of α(*t*) in SCI group between two postures. **(C)** Comparison of α(*t*) in the sitting position between two groups. **(D)** Comparisons of α(*t*) in the prone position between two groups.

Figure 7 presents the local scale exponents α(*t*) over healthy able-bodied control and SCI groups as means ±SE. In controls in both the sitting and prone postures, α(*t*) monotonically decreased with scale at the scales between 4 and 12 s and then showed a plateau (Figure 7A). At the scales shorter than 10 s, α(*t*) was significantly higher in the sitting posture than in the prone posture (Figure 7A), while at the scales larger than 15 s, α(*t*) remained at lower values for both postures. In subjects with SCI, α(*t*) slowly decreased at almost all scales. No differences were observed between two postures (Figure 7B). When comparing controls and subjects with SCI, in the sitting posture, α(*t*) in subjects with SCI was significantly lower at scales shorter than 8 s and tended to be higher at larger scales (Figure 7C). In the prone posture, α(*t*) at a narrow range of scales around 12 s was significantly higher in subjects with SCI than in controls (Figure 7D).

## Discussion

The main findings of this study are: (1) local scale exponent α(*t*) in healthy able-bodied controls rapidly decreased with scale at small scales in both the sitting and prone postures but slowly decreased in subjects with SCI (Figures 7A,B); (2) in the sitting posture, α(*t*) at small scales was lower in subjects with SCI than in controls (Figure 7C); and (3) in the prone posture, α(*t*) at moderate scales was higher in subjects with SCI than in controls. However, normalized low frequency (0.04–0.15 Hz) power, the ratio of low frequency power to high frequency (0.15–0.4 Hz) power and long-term (>11 beats) DFA coefficient α_2_ did not show significant difference between healthy controls and subjects with SCI in the prone posture (Table 2, Figure 6). Our findings support our hypothesis that α(*t*) can reveal important features of HRV in patients with SCI that are not reflected by the sympathovagal balance and DFA coefficients. This approach may be used to investigate the effects of SCI-induced autonomic damage on cardiovascular dysfunction.

Spectral analysis of HRV is considered to be a useful tool for evaluating cardiovascular autonomic control after SCI (Claydon and Krassioukov, 2008; Jan et al., 2013). In the present study, sympathovagal balance in controls increased in the sitting posture and decreased in the prone posture, whereas in patients with SCI, it showed only small changes (Table 2). These results were consistent with the previous studies (Claydon and Krassioukov, 2008; Jan et al., 2013). Increased sympathovagal balance in the sitting posture is associated with withdraw of vagal activity and enhanced sympathetic outflow to the heart (Wecht et al., 2006; Claydon and Krassioukov, 2008). In subjects with SCI, the increase in sympathovagal balance was attenuated, probably reflecting limited ability of vagal withdrawal to influence HR and reduced sympathetic cardiac modulation (Wecht et al., 2006). On the other hand, in the sitting posture, the short-term DFA coefficient α_1_ significantly increased in controls but not in subjects with SCI (Figure 6). These results were similar to those reported by Tulppo et al. (2001) and Merati et al. (2006). Our results suggested that the *LF* to *HF* because it has been demonstrated that ratio and DFA coefficients provide similar characterizations of HRV. This is not surprising, DFA coefficients can be obtained from the PSD (Willson et al., 2002). Willson et al. (2002) suggested that α_1_ is related to 2/[1 + (*HF*/*LF*)] and α_2_ is related to 2/[1 + (*LF*/*VLF*)] with *VLF* representing the power in the very low frequency region below 0.04 Hz. Platisa and Gal (2006) observed an approximated linear relationship between α_1_ and *ln*(*LF/HF*). Thus, it seems that α_1_ reflects the balance between the *LF* and *HF* oscillations and α_2_ reflects the balance between the *LF* and *VLF* oscillations.

Unlike DFA coefficients, local scale exponent reflects local properties of HRV. Castiglioni et al. (2011) suggested that local scale exponent reflects the local correlation properties of the data. By using autonomic blocking agents, they showed that the vagal outflow contributes with white-noise components to HRV while the cardiac sympathetic outflow adds Brownian motion components at short scales and contributes to a plateau between 40 and 80 s. In the present study, in able-bodied controls, α(*t*) at *t* between 4 and 12 s was significantly higher in the sitting posture than in the prone posture (Figure 7A). These scales correspond to frequencies between 0.083 and 0.25 Hz, which spread across the LF and HF bands. Supposing that the mean RRI is 0.8 s (see Table 2), the scales between 4 and 12 s correspond to 5–15 beats (Eq. 7). This range of beat number may spread across the ranges of observation window sizes over which α_1_ and α_2_ are calculated. As shown in Figure 6, α_2_ in controls in the sitting posture tended to be higher than in the prone posture but the differences did not reach a significant level. The reason might be that α_2_ was calculated over a wide range of observation window sizes, while in major parts of the range, α(*t*) exhibited similar behavior under two conditions (Figure 7A).

In the sitting posture, α(*t*) at *t* < 8 s was significantly lower in subjects with SCI than in controls (Figure 7C). The lower values of α(*t*) are compatible with lower values of α_1_ (Figure 6A) and lower values of sympathovagal balance (Table 2). At t between 6 and 15 s, roughly corresponding to 0.07–0.16 Hz, α(*t*) showed different curve configurations between two groups: it sharply decreased with t in controls but slowly decreased in subjects with SCI. The sharp decreasing trend in α(*t*) in controls suggests a more complex dynamics of HRV in controls. In contrast, the relative constant values of α(*t*) in subjects with SCI might reflect an attenuation of HRV. These features were not reflected by sympathovagal balance quantified by the spectral analysis and DFA coefficients.

In the prone posture, α(*t*) at t around 12 s was significantly higher in subjects with SCI than in controls (Figure 7D). The higher values of α(*t*) are compatible with the higher values of α_2_ in subjects with SCI although the differences in α_2_ between two groups did not reach the significant level (Figure 6B). The reasons for the higher values of α(*t*) and α_2_ in subjects with SCI are not clear. A possible explanation is the various levels and severities of SCI. It is well known that the mechanisms of HR regulation after SCI depend on the level and severity of injury (Claydon and Krassioukov, 2008). For example, it was reported that in the supine position, sympathovagal balance in controls was higher than in subjects with high-level lesions but lower than in those with low-level lesions (Merati et al., 2006; Claydon and Krassioukov, 2008). Accordingly, it was suggested that at rest, reduced sympathetic outflow are not balanced by reduced vagal outflow in subjects with cervical SCI and mechanisms underlying reduced vagal tone in subjects with thoracic SCI subjects are uncertain (Claydon and Krassioukov, 2008). Nevertheless, a striking phenomenon was that α(*t*) maintained relatively constant in subjects with SCI, whereas it monotonically decreased with scale in controls (Figure 7D). This might suggest that HRV in subjects with SCI was less complex compared to controls.

This study has several limitations. First, we only recruited 12 subjects with SCI into this study. However, our results confirmed our hypothesis that the spectrum of α(*t*) reveals important features of HRV in subjects with SCI that are not reflected by the spectral analysis (LF to HF ratio) and DFA coefficients. Second, we did not investigate the influences of injury level on α(*t*), because the C4–T5 group mainly consisted of incomplete spinal injury, while the T6–T12 group mainly consisted of complete spinal injury. Further studies may be required to determine the influences of injury level and completeness on α(*t*). Third, the SCI group was older compared to the control group. The age issue may affect the validity of our results. However, the age effect is a minor confounding variable when compared to postural changes (Jan et al., 2013). Further studies should consider using an age-matched research design.

### Conflict of interest statement

The authors declare that the research was conducted in the absence of any commercial or financial relationships that could be construed as a potential conflict of interest.
